# Racial and ethnic disparities in psychological care for individuals with FASD: a dis/ability studies and critical race theory perspective toward improving prevention, assessment/diagnosis, and intervention

**DOI:** 10.3389/fpubh.2024.1355802

**Published:** 2024-03-13

**Authors:** Madeline N. Rockhold, Blake A. Gimbel, Alesia A. Richardson, Carson Kautz-Turnbull, Emily L. Speybroeck, Erik de Water, Julianne Myers, Emily Hargrove, Maggie May, Samia S. Abdi, Christie L. M. Petrenko

**Affiliations:** ^1^Mt. Hope Family Center, University of Rochester, Rochester, NY, United States; ^2^Department of Pediatrics, University of Minnesota Twin Cities, Minneapolis, MN, United States; ^3^Great Lakes Neurobehavioral Center, Edina, MN, United States; ^4^ International Adult Leadership Collaborative of FASD Changemakers

**Keywords:** fetal alcohol spectrum disorder, FASD, prenatal alcohol exposure, Dis/Crit, disparities, race, ethnicity

## Abstract

Fetal alcohol spectrum disorders (FASD) are among the most common neurodevelopmental disorders and substantially impact public health. FASD can affect people of all races and ethnicities; however, there are important racial and ethnic disparities in alcohol-exposed pregnancy prevention, assessment and diagnosis of FASD, and interventions to support individuals with FASD and their families. In this article we use the Dis/Ability Studies and Critical Race Theory (Dis/Crit) framework to structure the exploration of disparities and possible solutions within these three areas (prevention, diagnosis, intervention). Dis/Crit provides a guide to understanding the intersection of dis/ability and race, while framing both as social constructs. Following the Dis/Crit framework, the systemic, historical, and contemporary racism and ableism present in psychological care is further discussed. We aim to elucidate these racial and ethnic disparities within the fields of psychology and neuropsychology through the Dis/Crit framework and provide potential points of action to reduce these disparities.

## Introduction

Fetal alcohol spectrum disorders (FASD) are neurodevelopmental conditions associated with prenatal alcohol exposure (PAE). In the United States, FASD affect approximately 1.1–5% of school-aged children (1), making them among the most common neurodevelopmental disorders. Diagnostic criteria for FASD include neurobehavioral differences in the presence of PAE and may include subtle facial features and smaller growth in body and brain size in some individuals (2, 3). Outcomes are variable in FASD; many risk and protective factors can influence functioning such as genetics, nutrition, receipt of services, and trauma/life stressors. Without adequate understanding and support, people with FASD are at higher risk for academic challenges (4), mental health conditions (5), housing and independent living issues, and trouble with the law (6, 7). FASD has a considerable public health impact (8) representing substantial societal and economic costs (9). Additionally, FASD are under-recognized and commonly misdiagnosed (10, 11).

Importantly, although FASD can affect people regardless of race, ethnicity, and socioeconomic status (SES), FASD is identified at higher rates in Native American, Black, and low-SES communities compared to White and middle/upper class communities (12). This pattern is the opposite in other neurodevelopmental disabilities such as attention-deficit/hyperactivity disorder (13, 14) and autism spectrum disorder (15, 16), with both diagnoses given to White individuals more frequently than Black, Indigenous, and People of Color (BIPOC) (13, 17). Greater attention to these disparities is needed within FASD and is relative to other neurodevelopmental disorders.

Dis/ability Studies and Critical Race Theory (Dis/Crit) provides a framework for understanding the intersections of race and dis/ability. We use the dis/ability notation instead of “disability” to disrupt the potentially harmful idea that some people cannot be successful within society’s view of appropriate functioning due to not being “able” (18, 19). Dis/Crit frames racial and dis/ability identities as social constructs. This means there are no clear biological indicators distinguishing unique races or dis/abilities. Society defines the boundaries of what constitutes different race and dis/ability groups, which change over time and are shaped by current and historical power structures and values. Critical to Dis/Crit is an appreciation of the influence of intersecting identities and how societal responses to individual differences result in multiple marginalization (20). Further, Dis/Crit acknowledges how this double marginalization is maintained by systems of oppression in ways that exacerbate inequality and injustice for BIPOC communities (21). For example, Black and Native American children, while overrepresented in special education (22), experience significant delays in diagnosis (and underdiagnosis) of neurodevelopmental conditions (17, 23).

**Figure 1:**
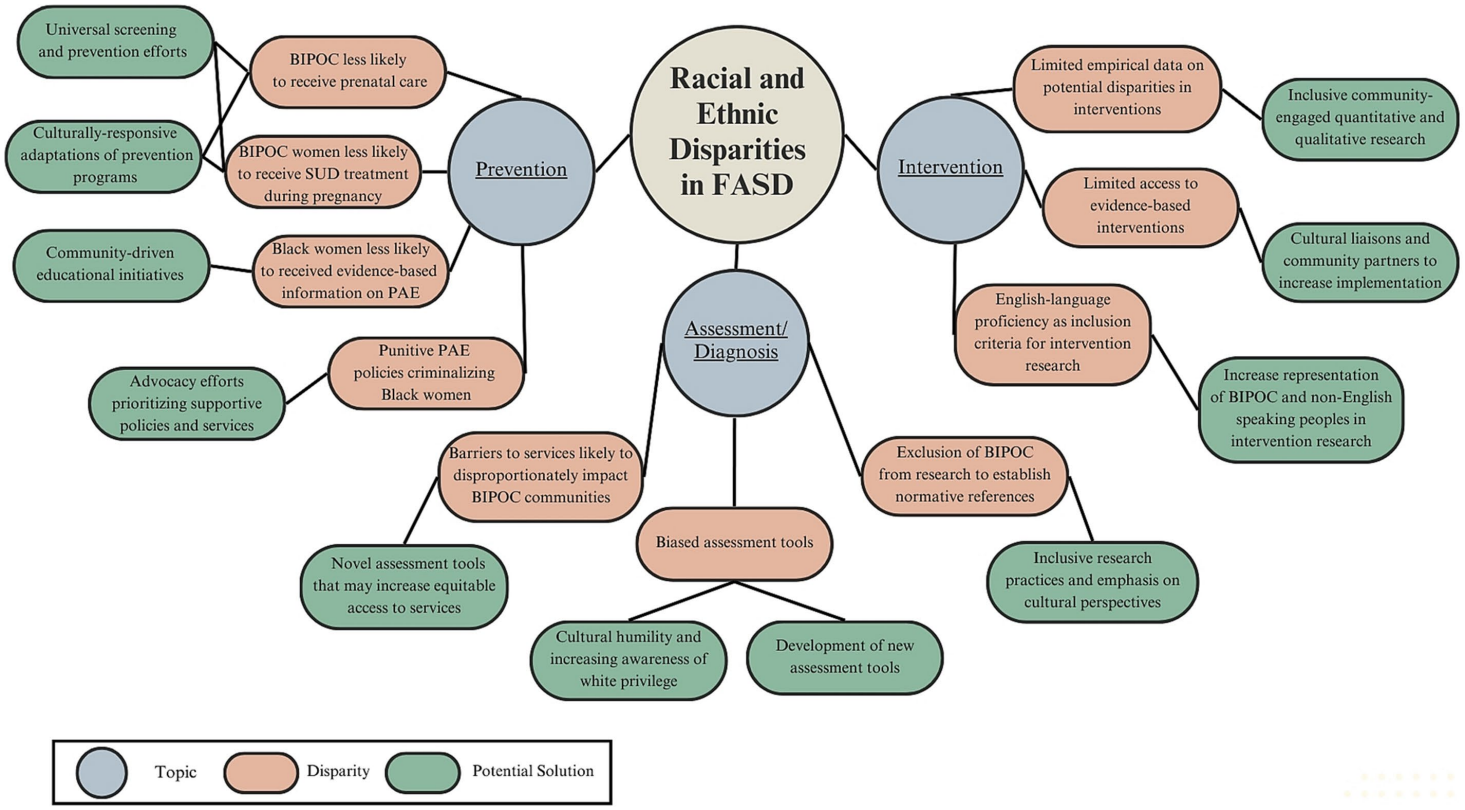
Graphical overview of racial and ethnic disparities in FASD within prevention, assessment/diagnosis, and interventions domains, and potential solutions.

Neurodevelopmental conditions result from a complex interplay of biological risks, social-historical influences, and environmental factors (24, 25). Dis/Crit provides a framework for understanding how racial/ethnic disparities in prevention, assessment/diagnosis, and intervention for individuals with FASD stem from complex interactions between social determinants of health and structural racism disproportionately affecting BIPOC (26). These issues represent an urgent public health crisis. Here, we provide examples of such disparities, perspectives regarding their potential causes and conditions, and solutions to advance equitable, culturally-responsive, and evidence-based psychological and neuropsychological care of individuals with FASD (Figure 1).

Our authorship team recognizes the importance of acknowledging our positionality. Our racial/ethnic backgrounds consist of individuals who are White, Black, Native American, and non-Hispanic. Multiple authors are also individuals with FASD. We acknowledge the power dynamics inherent in research, particularly within marginalized communities, and aim to approach this topic with cultural humility.

### Racial and ethnic disparities in prenatal care, substance use disorder treatment, and evidence-based information

Mitigating racial/ethnic disparities in FASD necessitates an initial focus on the prevention of alcohol-exposed pregnancies (AEPs). Racial/ethnic differences in AEP are understudied and findings have been equivocal. Several studies suggest an increased risk of AEPs in racial/ethnic minorities (27–30) whereas others suggest White individuals are more likely to have an AEP (31, 32).

Aligning with the Dis/Crit framework, it is imperative to recognize and address historical and ongoing racially-biased practices contributing to a disproportionate incidence of AEPs among BIPOC communities on a global scale. Examples of this include intergenerational trauma in Indigenous communities (33–35) and practices such as the “dop system” in South Africa, originating during apartheid, whereby White farm owners used alcohol as a form of labor payment and social control of marginalized racial groups (36–38).

Significant racial/ethnic disparities are evident in prenatal care, receipt of evidence-based AEP information, and interventions for substance and alcohol use disorders (SUDs and AUDs). Importantly, historical and ongoing race-based and intergenerational trauma impacts multiple non-White and BIPOC communities including Black/African Americans, American Indians and Alaska Natives, and people of Jewish and Asian ancestry (39). This historical context contributes higher rates of stress and disproportionate access to resources for marginalized groups (40). The prenatal period is a pivotal juncture for preventing AEPs (41). BIPOC women are less likely than White women to receive prenatal care (42) and experience disparities in respect and autonomy within healthcare settings (43) potentially contributing to underutilization of services (44). Regarding receipt of evidence-based prenatal education, the literature is mixed. Some research has suggested Black women are less likely to receive evidence-based information regarding the negative impacts of AEPs compared to White women (45). However, recent data indicate BIPOC and low-SES women are *more* likely to receive comprehensive prenatal health education (including AEP information) than White and economically advantaged counterparts despite experiencing greater risks for adverse birth outcomes suggesting such prenatal health education is inadequately addressing these disparities (46). Moreover, significant gaps in receipt of mental health treatment exist for Black and Hispanic pregnant women with SUDs when compared to White women, even after controlling for education level, income, age, health insurance, and urbanicity (47). Pregnant BIPOC with AUDs are less likely to seek treatment, partially due to risk of victimization stemming from policies pertaining to AEPs (48–50). These policies may heighten the risk of criminalization among BIPOC women and reduce reporting for fear of losing their children or other punitive measures (50).

#### Potential solutions

To reduce disparities in the receipt of evidence-based prenatal information and SUDs care, mental health providers can incorporate universal screening techniques, following the Screening, Brief Intervention, and Referral to Treatment (SBIRT) model (51). These efforts should include reflective and supervisory practice regarding racial/ethnic stereotyping potentially influencing disparities. Moreover, mental health practitioners can support open discussions about PAE by utilizing SAMHSA’s guidelines to reduce stigmatizing language (52) while being mindful of their respective state’s policies on AEPs and mandated child welfare reporting. Advocacy efforts should support policies prioritizing supportive over punitive actions for pregnant individuals who report an AEP. Efforts to reduce racial/ethnic disparities in AEPs should also involve tailored, culturally-responsive adaptations of prevention programs. Emphasis should be placed on collaboration with individuals with living experience (i.e., those with AEPs and BIPOC) as well as qualitative research methodology to understand experiences of intersectionality within systems of care. For example, Gonzales et al., emphasizes the importance of community-level healing practices (i.e., drumming, talking circles, and sharing practices related to pregnancy and parenting) to address racialized trauma as a key factor in contributing to AEPs in a Native American community (53). This underscores the need for culturally-informed, trauma-responsive training for mental health practitioners and those providing prenatal care. Finally, community-driven initiatives such as Proof Alliance’s program “Our Children Are Sacred” (54), which disseminates information on AEPs while addressing the historical trauma and racism experienced by Indigenous communities, will be important.

### Racial and ethnic disparities in assessment and diagnosis of FASD

The diagnostic process of FASD considers what is known about PAE; brain differences; smaller growth in height, weight, and head circumference; distinctive facial features (thin upper lip, smooth philtrum, short eye openings); and below average performance (i.e., 1.5–2 standard deviations below the mean depending on the diagnostic system) on norm-referenced neurocognitive and neurobehavioral measures (2, 55). Early diagnosis and intervention are crucial in supporting long-term functional outcomes (56, 57) and preventing adverse outcomes such as school disruption, mental health challenges, and trouble with the law, which have disproportionate consequences for BIPOC individuals. However, numerous barriers make early and accurate diagnosis a substantial hurdle for many individuals (58, 59). These barriers include a high cost of services, lack of healthcare access, lack of trained clinicians, the “hidden” nature of the condition for individuals who do not have facial and growth differences (25, 60), and stigma regarding AEPs (61–63). Such healthcare disparities disproportionately affect BIPOC, low-SES, and rural communities (64–66)—communities already at high risk for unequal access to prevention and early intervention. To our knowledge there are no empirical studies addressing potential ways in which race, dis/ability status, and other factors may contribute to healthcare inequalities for people with FASD and their families.

Psychologists and neuropsychologists play an important role in FASD diagnosis by collecting developmental histories, performing and interpreting neuropsychological assessments, and providing recommendations for services (67). The fields of psychology and neuropsychology have deep roots in structural racism. For example, many current cognitive performance assessments have been criticized for contributing to racially-biased educational placement of BIPOC and low-SES students in special education (68, 69).

Importantly, BIPOC have been systematically excluded from research aimed at determining normative references of typical development, which likely contributes to inequality in rates of FASD diagnosis (12). Normative references for lip/philtrum ratings are available only for children of European, Black/African American, and South African mixed-race heritage (70, 71). Although the three cardinal facial features associated with PAE are present across racial/ethnic groups, threshold cutoffs for diagnosis vary by race/ethnicity (71–73). Clinicians often have to use their judgment to decide which racial guide to use for individuals whose race is not represented in existing guides. Similarly, available normative data for head circumference and palpebral fissure length (eye openings) is based on smaller samples predominantly composed of individuals of European heritage (74, 75). Moreover, factors such as stereotype threat can lead to underestimation of cognitive abilities in BIPOC (76, 77) suggesting they may be more likely to meet cutoffs used to quantify below average performance in FASD diagnostic systems.

#### Potential solutions

First, it is imperative that we strive to improve representation and normative data for the tools we use to inform assessment and diagnosis of individuals of diverse racial/ethnic backgrounds (25, 73). Moreover, factors such as cultural, linguistic, and SES background; quality of and access to educational opportunities; test familiarity; stereotype threat; and appropriateness of test norms should be thoughtfully considered as potential contributors to observed performance on neuropsychological assessment (78). Use of technologies such as digital and mobile health could also increase access to assessment and diagnosis for families in rural and under-resourced communities (79), although similar attention is needed to possible normative limitations and content biases. Efforts to reduce racial/ethnic disparities in assessment and diagnosis of FASD must also include a concerted effort to address the systemic racism and White privilege ingrained in medicine (80, 81). White psychologists/neuropsychologists must acknowledge the “invisible knapsack” of White privilege (82) and its potential role in engendering a rational mistrust of medical providers for BIPOC. Aligned with the Dis/Crit framework, researchers and clinicians should also consider how ableist language (e.g., “impairment,” “deficit”) as used in current FASD diagnostic systems may contribute to stigmatization as well as the intersectionality of receiving an FASD diagnosis and identifying as BIPOC (18). Additionally, centering research efforts on understanding cultural perspectives of FASD (83), perspectives of individuals with living experiences (84), and how the intersection of race and dis/ability may further affect access to systems of care will be crucial to ensuring research outcomes are valuable to and address the priorities of the FASD community.

### Disparities in neurodevelopmental and behavioral interventions for people with FASD

Despite a considerably high prevalence, few interventions have been developed to support symptoms of FASD, and a majority of existing interventions have not yet progressed to active community implementation. A comprehensive review of the literature on interventions for FASD is beyond the scope of this paper (85, 86). To our knowledge, empirical studies on potential racial and ethnic disparities in interventions for FASD have not been published despite clear evidence for such disparities in neurodevelopmental disability literature more broadly (87–89). However, it is critical to acknowledge FASD intervention research and clinical services are rooted in the tradition of Western medicine, with limited access to services globally (85). Furthermore, numerous barriers impede evidence-based intervention for FASD across diverse cultures, including absence of local programs, housing of interventions in universities/medical centers who historically mistreated and oppressed BIPOC individuals, and failure of existing interventions to address individual differences at the intersection of dis/ability and culture (90).

A majority of interventions for FASD have been developed in North America (86) and many require English language proficiency as an inclusion criterion. While not specific to FASD intervention research, others have noted an increasing trend of English language requirements in behavioral clinical trials broadly (91). This constitutes a major limitation of current intervention research in the field of FASD likely to disproportionately affect BIPOC, non-English speaking, and bi/multilingual individuals and is likely to reduce the generalizability of intervention research. Many existing interventions are also caregiver-driven, which may hinder access for single-parent households or parents with multiple jobs.

#### Potential solutions

The inclusion of BIPOC and those with living experiences of FASD is crucial to community-engaged and inclusive intervention research (92). The use of qualitative methodologies to understand intersecting experiences of race and dis/ability in their entirety should be a first step in addressing disparities. Consistent with the Dis/Crit framework, it is imperative to include perspectives of individuals at the intersection of dis/ability and BIPOC identities as there are increased health disparities and unique experiences within systems for those holding such intersecting identities (93). Research advisory boards and community-based participatory research can be invaluable in ensuring the voices of community members are incorporated into ongoing research efforts (94–96). To highlight the need for community-engaged research, the annual meeting of the Fetal Alcohol Spectrum Disorder Study Group in 2022 featured a panel discussion including two adults with living experiences of FASD (“Nothing about us without us”) (97). A detailed qualitative analysis of best practices for community-engaged research with adults with FASD is currently under development (98).

Researchers should consider ways in which new interventions can be developed and implemented in partnership with communities to be person-centered, culturally-appropriate, and accessible in a variety of languages (85). Diverse and representative research teams that include BIPOC, individuals with FASD, and BIPOC individuals with FASD may also bridge these partnerships and provide valuable perspectives. Beyond community partnership, researchers should focus intervention outcomes on community members’ specific priorities and consider the complex ways race and cultural identities may intersect with such priorities. Cultural liaisons (e.g., trained interventionists/providers who are culturally-affiliated) may also be consulted to assist with implementing interventions and addressing cultural differences (90). Additionally, mobile health initiatives such as the Families Moving Forward Connect app and the My Health Coach app for adults with FASD (99–101), which focus on supporting parents and adults in managing health needs for individuals with FASD, may increase access to evidence-based information and facilitate community support networks. Careful research is planned to determine whether cultural adaptations would be beneficial. Lastly, strategies to increase BIPOC representation in research such as building community trust, employing equitable recruiting methods, and offering information in multiple languages (102, 103) will help address racial/ethnic disparities in FASD intervention work. Implementation science guidelines addressing structural racism and health disparities can also guide scientifically sound intervention work (104, 105).

## Discussion

A number of important factors have contributed to emerging evidence for racial/ethnic disparities in psychological care for individuals with FASD. While our understanding is far from complete, Dis/Crit provides a useful framework for conceptualizing the causes and conditions of such disparities and informing a path forward (21). In addition to the specific recommendations for potential solutions outlined above, several broad approaches will support systems-level changes coordinated across disciplines and settings.

It is of utmost importance to include individuals with living experiences of dis/ability (including FASD) and BIPOC at all levels of research aimed at understanding and supporting individuals with FASD. Community-engaged research practices are needed to foster collaborative dialogues with community members and highlight their cultural knowledge and living-experiences to better understand disparities within these communities. Moreover, community-engagement would support equitable research processes that empower individuals with FASD rather than further stigmatize/marginalize them. For example, adults with FASD who are authors on the current paper suggest a future aim of understanding how religion ties into cultural differences for people with FASD and how this may impact the diagnosis and intervention. Researchers and clinicians must strive for inclusivity, cultural humility, and equitable access to resources so individuals with FASD and their communities are supported in navigating each stage from prevention to intervention regardless of race/ethnicity. Equally important is diversification of the (predominantly White) psychology workforce in the United States (106, 107), which will allow for individuals seeking clinical services to feel represented and understood, help in reducing language barriers, and potentially lead to increased services globally. Importantly, communication and collaboration across systems of care (i.e., medical professionals and educators) is crucial in reducing disparities and promoting early and accurate identification of FASD. As primary care providers and other professionals (e.g., educators) are often at the forefront of care, psychologists and mental health professionals could offer support on early detection of AEP and FASD, provide referral resources, and intervention. Lastly, further research is needed to understand cultural perceptions and understanding of FASD (108), which could potentially increase stigmatization and create additional barriers to care for individuals with FASD (109–111). In addition to understanding cultural perceptions of the challenges of individuals with living experience of FASD and their families/caregivers, it will be important to focus on the strengths and resilience of the FASD community (112, 113).

## Conclusion

In sum, racial/ethnic disparities in psychological care for individuals with FASD represent important challenges to the field and will require coordinated, collaborative, and inclusive efforts to overcome. We hope that increased awareness will foster discussion and stimulate creative solutions to improve equitable, culturally-responsive, and evidence-based psychological services and support for individuals with FASD and their families.

## Data availability statement

The original contributions presented in the study are included in the article/supplementary material, further inquiries can be directed to the corresponding author.

## Author contributions

MR: Conceptualization, Visualization, Writing – original draft, Writing – review & editing. BG: Visualization, Writing – original draft, Writing – review & editing. AR: Writing – original draft, Writing – review & editing. CK-T: Writing – original draft, Writing – review & editing. ES: Writing – original draft, Writing – review & editing. EW: Writing – original draft, Writing – review & editing. JM: Writing – original draft, Writing – review & editing. EH: Writing – original draft, Writing – review & editing. MM: Writing – original draft, Writing – review & editing. SA: Writing – original draft, Writing – review & editing. CP: Supervision, Writing – original draft, Writing – review & editing.
